# Smoking Status and Cessation Counseling Practices Among Physicians, Guangxi, China, 2007

**Published:** 2009-12-15

**Authors:** Abu S. Abdullah, Jiatong Zhou, Dongmei Huang, Songyi Lu, Shuiying Luo, Vivian C. Pun

**Affiliations:** Department of International Health, Boston University School of Public Health; Center for Disease Control and Prevention, Nanning, Guangxi, China; Center for Disease Control and Prevention, Nanning, Guangxi, China; Center for Disease Control and Prevention, Nanning, Guangxi, China; Center for Disease Control and Prevention, Nanning, Guangxi, China; Boston University School of Public Health, Boston, Massachusetts

## Abstract

**Introduction:**

We examined Chinese physicians' smoking behavior, knowledge of smoking's health effects, and compliance with accepted cessation counseling practices.

**Methods:**

We used a structured questionnaire adapted from the Global Health Professionals Survey of the World Health Organization to survey Chinese physicians based at 5 hospitals in Nanning, Guangxi Province, China.

**Results:**

The response rate was 85% for a total of 673 completed questionnaires. Of the 673 respondents, 73% were men, 42% were aged 30 years or younger, and 26% were smokers (men, 35%; women, 3%). Only 28% of the smokers were ready to quit immediately. A substantial proportion of physicians did not have adequate knowledge of smoking-related health hazards or favorable attitudes toward smoking cessation counseling. Asking patients whether they smoked and recording smoking status in the medical record were significantly associated with being female and being very well or somewhat prepared to counsel patients about smoking cessation. Advising patients to quit smoking was significantly associated with being female, being a nonsmoker, being very well or somewhat prepared to counsel patients about smoking cessation, and having read any smoking cessation guidelines.

**Conclusions:**

Our findings suggest that smoking is common among male Chinese physicians and that Chinese physicians have inadequate knowledge of smoking's health hazards and of how to help smokers quit. Physicians in China and their patients who smoke would benefit from widely accessible Chinese clinical practice guidelines on smoking cessation, better medical school education about the health risks of smoking, and government funding of cessation medications.

## Introduction

Approximately one-third of the world's smokers reside in China (1), where the national smoking prevalence is 31% (men, 57%; women, 3%) (2,3). Nearly 800,000 Chinese die each year as a result of smoking (4), and the number will increase to 2 million by 2025 if current smoking rates continue (5). Smoking is expected to cause one-third of all deaths among Chinese men by 2030 (6).

Medical interventions can be effective in helping smokers to quit (7). Not only are physicians primarily responsible for delivering such interventions, they are usually viewed as role models for health-related behavior such as smoking (7). Although the dangers of smoking are well known throughout the medical profession, physicians do not always set a good example for patients. The World Health Organization (WHO) documents that the prevalence of smoking among physicians in China is 61% for men and 12% for women (1). Other studies have reported different prevalence rates. For example, a study of 3,553 physicians from 6 Chinese cities reported a smoking prevalence of 23% (men, 41%; women, 1%) (8). In another study of 786 physicians, overall smoking prevalence was 20% (men 54%, women 3%) (9). Annually, 76% of smokers are seen by physicians in China (10), creating an opportunity to counsel patients about quitting. However, few physicians ask about smoking status or advise smokers to quit. In a study by Jiang et al, only 48% of physicians asked about patients' smoking status, and 64% advised smokers to quit (11). Other studies showed that less than half of physicians "often" or "always" advise smokers to quit (12-14). The reasons for not asking about smoking status or advising smokers to quit have not been well documented for Chinese physicians. We examined the smoking status of physicians and their knowledge, attitudes, and confidence about helping their patients who smoke to quit. We also studied how well their cessation counseling practices conformed to a US clinical practice guideline (15).

## Methods

A cross-sectional survey of physicians was conducted in 5 hospitals in Nanning, Guangxi Province, from March 5 through March 15, 2007. The 5 hospitals were the Number One People's Hospital of Nanning, the Number One and the Number Two Affiliated Hospitals of Guangxi Traditional Chinese Medical College, the Number One Affiliated Hospital of Guangxi Medical University, and the Guangxi Comprehensive Hospital. All physicians who worked in the departments of internal medicine, surgery, pediatrics, and gynecology were included in the survey. We selected these departments to maintain uniformity among the hospitals, because all the selected hospitals had these departments and they accounted for 70% of the physicians in the hospitals. The study was approved by the institutional review boards of the Chinese Center for Disease Control and Prevention, Guangxi. Because the survey was anonymous, study participants did not sign consent forms.

A 77-item self-administered questionnaire was developed with reference to the Global Health Professionals Survey (16) and a Hong Kong physicians' survey (17) and was translated into Chinese. The following sections from the Hong Kong questionnaire were added to the 53-item Global Health Professionals Survey: confidence level (4 questions), attitudes toward smoking cessation guidelines (3 questions), training received on cessation counseling (7 questions), worksite practices (6 questions), and use of drug therapy to help smokers quit (4 questions). We translated from the English version to the Chinese version and then back-translated to English before conducting a pilot test with 20 participants. The questionnaire was delivered to physicians in the selected departments through the hospital director's office and collected in the same office in a sealed envelope.

Besides demographic information, the questionnaire asked about knowledge of the health hazards of smoking and attitudes toward smoking and cessation counseling. A Likert scoring system was used to categorize the responses as 1 for "strongly agree" through 5 for "strongly disagree." The survey assessed the degree of preparedness to counsel patients on smoking cessation, and responses were categorized as 1 for "very well prepared," 2 for "somewhat prepared," and 3 for "not at all prepared." Respondents answered yes or no to questions on smoking status, training in cessation counseling, and usual practices in cessation counseling.

Respondents were grouped as current, former, or never smokers according to WHO classification (18). Current smokers were defined as those who had smoked for at least 6 months during their lifetime and were smoking at the time of the survey. Former smokers were defined as those who had smoked for at least 6 months during their lifetime but had not smoked for at least 6 months before the survey. Never smokers were defined as those who had never smoked or had smoked for fewer than 6 months during their lifetime. For this report, former and never smokers were collapsed into the category "nonsmokers."

Data were analyzed by using SPSS for Windows version 11.0 (SPSS, Inc, Chicago, Illinois). We used χ^2^ tests and multivariate analysis to assess the relationships between variables. Significance was established as *P* < .05 (2-tailed).

## Results

### Characteristics of the respondents

Of 792 surveys delivered, 673 were completed, a response rate of 85%. Response rates were almost identical in all the departments. Of the respondents, 73% were men, 42% were aged 30 years or younger, and more than 68% specialized in internal medicine or surgery (Table 1). The prevalence of smoking was 26%. Fifty percent reported that no smoke-free policy was in place in their hospital, 93% had not received any training on smoking cessation counseling, and 62% had not read any smoking cessation guidelines.

Male physicians reported a much higher rate of smoking than did female physicians (35% vs 3%) (Table 2). The highest rate was seen among physicians aged 41-50 years (38%). When asked about their readiness to quit smoking, only 28% of smokers said they were ready to quit immediately, whereas 25% were thinking about quitting within 6 months, and 47% were not ready to quit within the next 6 months.

Most respondents (99%) knew that smoking is harmful to health (Table 3). More than 80% knew that secondhand smoke increases the risk of lung and heart diseases in nonsmoking adults and the risk of lower respiratory tract illnesses such as pneumonia in exposed children. Nonsmokers were more likely to have such knowledge than smokers (*P* = .002). Only 49% of physicians knew that neonatal death is associated with secondhand smoke, and less than 40% knew that nicotine replacement therapy and the antidepressant bupropion can help people to quit smoking.

Eighty percent of respondents believed that health professionals should serve as nonsmoking role models for their patients and the public and that they should routinely advise smokers to avoid smoking around children (Table 3). Most physicians (66%) believed that their patients' likelihood of quitting would be better if a health professional advised them to quit, and 54% believed that health professionals who smoke are less likely to advise people to stop smoking. More than 90% believed that health professionals should routinely ask about patients' smoking habits. Most respondents (92%) believed hospitals and health care centers should be smoke-free. Smokers were less likely than nonsmokers to believe in these smoke-free policies (*P* < .001).

Only 58% of all physicians believed that they could explain the risks attributed to smoking in detail to patients (Table 3). Smokers were less likely than nonsmokers to have such confidence (40% vs 64%, *P* < .001). Even fewer (24%) believed that their knowledge was sufficient for helping patients to stop smoking, and only 16% believed that their skills were sufficient for helping patients to stop smoking. Less than 6% believed that they knew how to prescribe medication to treat tobacco dependence or that they could assess a smoker's level of nicotine dependence by using the Fagerström Test for Nicotine Dependence.

Of the 673 respondents, 479 (71%) usually asked every patient about their smoking status, 362 (54%) recorded smoking status in the medical record, and 525 (78%) advised smokers to quit (Table 4). A higher proportion of female than male physicians asked about smoking and advised patients to quit (*P* = .04). A higher proportion of nonsmokers (80%) than smokers (63%) advised patients to quit (*P* < .001). More physicians who worked in hospitals without a smoke-free policy and who were prepared to counsel patients on how to quit followed all 3 smoking cessation practices (asking about smoking status, recording smoking status in the medical record, and advising patients to quit) (*P* < .001). More physicians who had read any smoking cessation guidelines recorded smoking status and advised patients to quit (*P* = .03).

Multivariate analysis showed that asking about smoking was significantly associated with physicians being female, working in a hospital without a smoke-free policy, and being prepared for smoking cessation counseling (Table 5). Recording patients' smoking status was significantly associated with being female, working in a hospital without a smoke-free policy, being prepared for smoking cessation counseling, and having read any smoking cessation guidelines. Advising patients to quit was significantly associated with being a nonsmoker, working in hospital without a smoke-free policy, being prepared for smoking cessation counseling, and having read any smoking cessation guidelines.

## Discussion

We found that the smoking rate among physicians in Nanning was 26%, which is consistent with previous studies (8,12). Reported smoking rates range from 13% (19) to 57% (20); such a wide range may be partially explained by the sociocultural and geographic variations in China, the year in which the study took place, or the specialty of the physicians. Our smoking rate is lower than those of observational studies conducted among physicians in the Netherlands (38%) (21), Japan (34%) (22), and France (32%) (23), but higher than those reported for the United States (3%) (24), New Zealand (5%) (25), and the United Kingdom (7%) (26). Our rate is also much higher than that found for Chinese physicians in Hong Kong (4%), although the response rate in the Hong Kong study was low (19%) (17). We found that smoking is more prevalent among male physicians than among female physicians, which reflects the smoking pattern among China's general population; nevertheless, overall prevalence among physicians is much lower (8). When compared with population data from WHO, the prevalence rate among male physician respondents to our survey was approximately half that of the general population (67%) (3).

Li et al argued that cigarette smoking is a "universal phenomenon" (14), and it is deeply entrenched in Chinese society (27). Because our study showed that physicians who smoke are less likely to believe that health professionals should serve as nonsmoker role models for their patients and the public, we suggest that interventions aimed at reducing cigarette smoking among physicians would lower the overall smoking rate.

We found that Chinese physicians lack sufficient knowledge about the health hazards of smoking. They need more education about the link between maternal smoking and neonatal death (8). In addition, physicians, especially those who smoke, need more education about the links between secondhand smoke and risk of lung disease, heart disease, and lower respiratory tract disease. Incorporating and adapting best practices for counseling patients about cessation into the medical school curriculum may increase knowledge and prevent smoking in young medical students (5,28).

We found that physicians' preparedness for smoking cessation counseling was associated with good smoking cessation practices (ie, ask, record, and advise), which indicates the need for professional training; Chinese physicians lacked expertise and familiarity with professional guidelines on how to provide smoking cessation counseling. Finding ways to surmount these barriers should be a priority in promoting tobacco control activities by health care professionals in China (15).

The finding that working in a hospital with smoke-free policies was negatively associated with cessation counseling was counterintuitive. In follow-up interviews with key hospital personnel and physicians, we learned that hospitals with smoke-free policies often did not enforce them. In addition, many physicians in this follow-up did not know whether their hospital was smoke-free. We suggest that smoke-free policies need to be better communicated and enforced.

We observed that younger physicians were more likely to follow good cessation counseling practices. Counseling practices may, therefore, improve with time. Continuing education on smoking cessation should be offered to all age groups of physicians, and clinical staff should routinely assess and record the smoking status of every patient in the hospital's medical record systems as a vital sign (15).

Asking about smoking (71%) was less common than advising to quit smoking (78%) among the physicians. Although it is consistent with another study among the physicians in China (11), it is in contrast with studies among the US (29) and Hong Kong (26) physicians. US physicians ask about smoking status during two thirds of all visits, but only advise about 20% of smokers to quit (29). Similarly, 77% of the Hong Kong physicians usually ask about smoking and only 29% advise smokers to quit. The higher rate of advising to quit than asking about smoking in the current study may be due to the fact that patients raise the issue themselves because of  increased awareness. As many physicians smoke they are reluctant to proactively ask about smoking status of the patients. Also, it is possible that physicians who did advise smokers to quit wished to encourage patients to buy cessation medications. Cessation medications are not subsidized by the health care system; smokers have to buy these medications with their own money. In many cases, physicians get incentives from pharmaceutical companies for prescribing branded medications which might have encouraged physicians to advise their patients to quit and, probably, to suggest buying certain cessation products. In contrast, asking and recording smoking status is not mandatory in the Chinese health care system and there is no incentive for doing so. Furthermore, we found that advising smokers to quit was higher in our survey than the 64% reported by a study of Chinese physicians in another region (11). Our higher prevalence of giving quitting advice may be due to the regional variations in smoking patterns and implementation of smoke-free polices or to the presence of more never smokers (69%) in our sample compared with the other study (55%) (11). In this study, former or never smokers were more likely than current smokers to advise smokers to quit, which is consistent with the findings in another study (30).

Reading any guidelines about counseling patients to quit smoking was positively associated with recording smoking status and advising smokers to quit. However, most guidelines were available in English, but most Chinese physicians cannot read English. A brief Chinese language guideline was developed in 2007 (written communication, Dr Jiang Yuan, Director of the National Tobacco Control Office, China CDC, Beijing, China, June 2008), after our study was completed, and is not yet available to all physicians. In a follow-up investigation, we found that most physicians were not aware of this brief Chinese guideline. However, many had heard about the international guidelines, mostly US and UK guidelines, and a few had briefly investigated these international guidelines through the Internet, but did not understand the details of the recommendations because of the language barriers. We believe that developing a national guideline in Chinese and promoting it to the physicians would be useful.

Some limitations of this study should be noted. First, the response rate of 85% may have been higher if we had delivered a second survey. Second, because the survey was anonymous, no information on the characteristics of nonresponding physicians is available, and respondents may have differed from nonrespondents, which may limit the generalizability of the study findings. Finally, respondents may have underreported behaviors viewed as deviant or socially undesirable (31).

In conclusion, our study uncovered a lack of knowledge about the health effects of smoking, a less than favorable attitude toward counseling smokers to quit, and a lack of confidence in providing such counseling among Chinese physicians. A large proportion of physicians did not routinely ask their patients about smoking status, record smoking status, or counsel patients about how to quit. Thus, a Chinese-language cessation guideline that emphasizes the importance of counseling smokers to quit and how to deliver that counseling should be available, along with training on how to implement the guideline. Responses to the questions about knowledge, attitudes, and confidence to deliver smoking cessation counseling (ask, record, and advise) reveal factors that may influence physicians to help their patients quit smoking and to quit smoking themselves. These findings are potentially generalizable to other areas with cultural and development similarities that have not yet established smoking cessation services as a norm in the clinical setting. However, the generalizability of the findings to physicians outside of Nanning, Guangxi province, should be considered with caution. Overall, our findings stress the need to enforce smoke-free hospital policies and develop interventions that will help physicians to quit smoking.

## Figures and Tables

**Table 1:** Characteristics of Respondents, Survey on Smoking Status and Cessation Counseling Practices Among Physicians, Guangxi, China, 2007

**Characteristics**	Respondents, No. (%)^a ^(N = 673)
**Male sex**	493 (73)
**Age, y**	
20-30	283 (42)
31-40	240 (36)
41-50	98 (15)
>50	52 (8)
**Specialty**	
Internal medicine	250 (37)
Surgery	210 (31)
Pediatrics	120 (18)
Gynecology	80 (12)
Others	13 (2)
**Smoking status**	
Current smoker	176 (26)
Former smoker	32 (5)
Never smoker	465 (69)
**Have smoke-free policy in the hospital**	337 (50)
**Received formal training in smoking cessation**	44 (7)
**Have read any form of smoking cessation guidelines**	256 (38)

a Percentages may not total 100 because of rounding.

**Table 2:** Smoking Prevalence Among 673 Physicians, Guangxi, China, 2007

Characteristic	Current Smoker, No. (%)^a^	Nonsmoker, No. (%)^b^	*P* Value^c^
**Sex**			
Male	170 (35)	323 (66)	<.001
Female	6 (3)	174 (97)	
**Age, y**			
20-30	61 (22)	222 (78)	.003
31-40	70 (29)	170 (71)	
41-50	37 (38)	61 (62)	
>50	8 (15)	44 (85)	

a Percentages may not total 100 because of rounding.

b Nonsmokers include former smokers and never smokers.

c *P* values derived from χ^2^ analysis.

**Table 3:** Knowledge, Attitudes, and Confidence to Deliver Cessation Counseling Among 673 Physicians, Guangxi, China, 2007

**Statements**	All, No. (%)	Current Smoker, No. (%) (n = 176)	Nonsmoker,^a^ No. (%) (n = 497)	*P* Value
**Knowledge ("strongly agree" or "agree")**				
Smoking is harmful to your health.	669 (99)	153 (87)	486 (98)	<.001
Neonatal death is associated with passive smoking.	330 (49)	79 (45)	251 (51)	.35
Maternal smoking during pregnancy increases the risk of sudden infant death syndrome.	517 (77)	128 (72)	389 (78)	.33
Passive smoking increases the risk of lung disease in nonsmoking adults.	600 (89)	137 (78)	463 (93)	<.001
Passive smoking increases the risk of heart disease in nonsmoking adults.	548 (81)	123 (70)	425 (86)	<.001
Passive smoking increases the risk of lower respiratory tract illnesses such as pneumonia in exposed children.	576 (86)	137 (78)	439 (88)	.002
Nicotine replacement therapy (eg, patch, gum, inhaler) can improve smokers' chance of stopping.	257 (38)	55 (31)	202 (41)	.08
Bupropion (ie, Zyban) is effective in helping people quit smoking.	228 (34)	52 (30)	176 (35)	.07
**Attitudes ("strongly agree" or "agree")**				
Health professionals serve as role models for their patients and the public.	538 (80)	120 (68)	418 (84)	<.001
Health professionals should set a good example by not smoking.	536 (80)	114 (65)	422 (85)	<.001
Patients' chances of quitting smoking are increased if a health professional advises them to quit.	443 (66)	101 (57)	342 (69)	.01
Health professionals should routinely ask about their patients smoking habits.	613 (91)	158 (90)	455 (92)	.70
Health professionals should routinely advise their smoking patients to quit smoking.	445 (66)	110 (63)	335 (67)	.49
Health professionals who smoke are less likely to advise people to stop smoking.	365 (54)	71 (40)	294 (59)	<.001
Smoking in enclosed public places should be prohibited.	629 (94)	158 (90)	471 (95)	.07
Hospitals and health care centers should be "smoke-free."	621 (92)	148 (84)	473 (95)	<.001
NRT should be made available by the Chinese health care system.	256 (38)	59 (34)	197 (40)	.29
Health professionals should routinely advise patients who smoke to avoid smoking around children.	534 (79)	126 (72)	408 (82)	.002
**Confidence level ("very well prepared" or "somewhat prepared")^b^**				
My current knowledge is sufficient for helping patients to stop smoking.	163 (24)	32 (18)	131 (26)	.05
I can explain the risks attributed to smoking in detail to patients.	389 (58)	70 (40)	319 (64)	<.001
My current skills are sufficient for helping patients to stop smoking.	108 (16)	21 (12)	87 (18)	.05
I know how to prescribe medication (NRT or bupropion) to treat tobacco dependency.	40 (6)	7 (4)	33 (7)	.15
I can assess a smoker's different stages of readiness to quit.	56 (8)	17 (10)	39 (8)	.71
I can assess a smoker's level of nicotine dependence using the Fagerström Score.^c^	33 (5)	8 (5)	25 (5)	.92
I can help a smoker to quit even if the smoker thinks that it is difficult to give up.	82 (12)	22 (13)	60 (12)	.48

Abbreviation: NRT, nicotine replacement therapy.

a Nonsmokers include former smokers and never smokers.

b Refers to confidence in counseling patients about smoking cessation.

c The Fagerström Test for Nicotine Dependence (http://www.wfts.org/programs_and_services/images/Fagerstrom_Test.pdf.).

**Table 4:** Comparison of Cessation Counseling Practices Among 673 Physicians, Guangxi, China, 2007

Characteristic	Ask About Smoking		Record Smoking Status		Advise Patients to Quit	
	No. (%) (n = 479)	*P* Value	No. (%) (n = 362)	*P* Value	No. (%) (n = 525)	*P* Value
**Age, y**						
20-30	234 (83)	<.001	164 (58.0)	<.001	221 (78)	.23
31-40	171 (71)		138 (58)		193 (80)	
41-50	58 (59)		45 (46)		76 (78)	
>50	16 (31)		15 (29)		35 (67)	
**Sex**						
Male	340 (69)	.04	256 (52)	.11	370 (75)	.002
Female	139 (77)		106 (59)		155 (86)	
**Smoking status**						
Current smoker	69 (76)	.29	49 (54)	.99	57 (63)	<.001
Nonsmoker	410 (71)		313 (54)		468 (80)	
**Have smoke-free policy in the hospital**						
Yes	225 (67)	.02	160 (48)	.002	242 (72)	<.001
No	254 (75)		202 (60)		283 (84)	
**Degree of preparedness**						
Very well prepared or somewhat prepared	258 (77)	<.001	197 (59)	.007	296 (89)	<.001
Not at all prepared	221 (65)		165 (49)		229 (68)	
**Training in smoking cessation**						
Yes	36 (82)	.11	23 (52)	.83	34 (77)	.90
No	443 (70)		339 (54)		491 (78)	
**Have read any smoking cessation guidelines**						
Yes	191 (74)	.16	156 (61)	.005	211 (82)	.03
No	288 (69)		206 (50)		313 (75)	

**Table 5:** Odds of Engaging in Cessation Counseling Practices Among 673 Physicians, Guangxi, China, 2007

Characteristic	Ask About Smoking		Record Smoking Status		Advise Patients to Quit	
	OR^a^ (95% CI)	*P* Value	OR^a^ (95% CI)	*P* Value	OR^a^ (95% CI)	*P* Value
**Sex**						
Male	0.66 (0.44-0.98)	.04	0.66 (0.44-0.98)	.04	0.75 (0.53-1.07)	.11
Female	1 [Reference]		1 [Reference]		1 [Reference]	
**Smoking status**						
Current smoker	1.32 (0.79-2.20)	.29	1.00 (0.64-1.56)	.99	0.41 (0.25-0.65)	<.001
Nonsmoker	1 [Reference]		1 [Reference]		1 [Reference]	
**Have smoke-free policy in the hospital**						
Yes	0.66 (0.47-0.93)	.02	0.62 (0.45-0.84)	.002	0.49 (0.33-0.72)	<.001
No	1 [Reference]		1 [Reference]		1 [Reference]	
**Degree of preparedness**						
Very well or somewhat prepared	1.81 (1.29- 2.55)	<.001	1.52 (1.12-2.06)	.01	3.74 (2.49-5.62)	<.001
Not at all prepared	1 [Reference]		1 [Reference]		1 [Reference]	
**Training in smoking cessation**						
Yes	1.89 (0.86-4.14)	.11	0.94 (0.51-1.73)	.83	0.96 (0.46-1.98)	.90
No	1 [Reference]		1 [Reference]		1 [Reference]	
**Have read any smoking cessation guidelines**						
Yes	1.29 (0.91-1.82)	.16	1.57 (1.15-2.16)	.005	1.54 (1.04-2.28)	.03
No	1 [Reference]		1 [Reference]		1 [Reference]	

Abbreviations: OR, odds ratio; CI, confidence interval.

a Adjusted odds ratios for age and each characteristic.
